# Ethnic Differences in Bone Microarchitecture

**DOI:** 10.1007/s11914-020-00642-y

**Published:** 2020-11-17

**Authors:** Ruth Durdin, Camille M Parsons, Elaine Dennison, Nicholas C Harvey, Cyrus Cooper, Kate Ward

**Affiliations:** 1 MRC Lifecourse Epidemiology Unit, University of Southampton, Southampton General Hospital, Southampton, SO16 6YD UK; 2 National Institute for Health Research Biomedical Research Centre, University of Southampton and University Hospital Southampton NHS Foundation Trust, Southampton, UK; 3 National Institute for Health Research Musculoskeletal Biomedical Research Unit, University of Oxford, Oxford, UK

**Keywords:** Adult, Microarchitecture, Quantitative computed tomography, Dual-energy X-ray absorptiometry, Ethnicity

## Abstract

**Purpose of the Review:**

The aim of this review is to briefly introduce updates in global fracture epidemiology and then to highlight recent contributions to understanding ethnic differences in bone density, geometry and microarchitecture and consider how these might contribute to differences in fracture risk. The review focuses on studies using peripheral quantitative computed tomography techniques.

**Recent Findings:**

Recent studies have contributed to our understanding of the differences in fracture incidence both between countries, as well as between ethnic groups living within the same country. In terms of understanding the reasons for ethnic differences in fracture incidence, advanced imaging techniques continue to increase our understanding, though there remain relatively few studies.

**Summary:**

It is a priority to continue to understand the epidemiology, and changes in the patterns of, fracture, as well as the underlying phenotypic and biological reasons for the ethnic differences which are observed.

## Introduction

Demographic shifts towards older populations are occurring in countries worldwide [1]. Across the globe, osteoporotic fractures remain a substantial healthcare burden; in order to prevent fractures and therefore lessen their associated burden, it is important to understand why these differences exist. The risk of fragility fracture is underpinned by low bone mineral density, but past evidence shows other aspects of bone strength also contribute to fracture risk. The aim of this review is therefore to present updates on global, and ethnic, patterns of fracture incidence and to describe ethnic differences in bone microarchitecture, exploring the potential underlying bone phenotype for these differences.^1^

## Global and Ethnic Variation in Fracture Incidence

Worldwide, there is an approximately 10-fold range in hip fracture incidence rates [2]. For women, countries with the highest annual age-standardised hip fracture incidences included Denmark, Sweden and Austria; the lowest included Morocco, Ecuador and Tunisia. Recent additions to the global literature include data reporting sex-specific trends in hip fracture incidence rate in Lebanon between 2006 and 2017, where a decrease in fracture incidence was observed over follow-up [3]. There remains a gap in data in low- and middle-income countries (LMICs^2^), which are discussed later [3, 4••, 5].

While the data presented above demonstrate between-country differences in fracture incidence, variation in fracture rates between ethnic groups in the same country has also been described [6]. For instance, in the UK, notable ethnic differences in hip fracture were recently shown using data from the Clinical Practice Research Datalink (CPRD); fragility fracture rates (radius/ulna, spine and femur/hip fractures) were the highest in White, and the lowest in Black, men and women aged 50 years and above [6]. In Singapore, age-standardised hip fracture incidence rates decreased between 2000 and 2017 [7]. Hip fracture incidence rates were higher in women than men and ethnic differences, examined in women only, existed; Chinese women had the highest fracture rates, compared to Malay and Indian women, yet were also the only ethnic group in which a decline in rate was during seen during the follow-up period [7].

## Mortality Post Hip Fracture

In England, ethnic differences in mortality within 1 year of a fracture, including a higher mortality rate in Black women compared to White women, have been demonstrated using data from the CPRD [8]. Both fracture, such as hospital-related, and non-fracture related, such as socioeconomic status, reasons are suggested for these ethnic differences in outcome. In a study of universally insured health patients in the USA, the odds ratio (adjusted for covariates including socioeconomic status) of 1-year mortality following hip fracture surgery was significantly lower in Hispanic and Asian patients, but similar in Black patients compared to White patients [9]. Finally, in relation to hip fracture care, Black patients in the USA have been shown to be at greater risk of aspects such as delayed surgery and readmission after surgery compared to White patients [10]. Of note, evidence from this study also indicated differences in hip fracture care according to health insurance type. The multifaceted reasons for these ethnic disparities certainly require further investigation to be able to reduce the disparities in outcomes which exist.

## Rising Burden of Fracture in Low- and Middle-Income Countries

Globally, the rising ageing population and burden of non-communicable diseases including osteoporosis associated with ageing are a concern; however, in LMICs, this is in conjunction with the existing burden of communicable diseases, such as HIV. The older peoples’ health is also affected by other issues such as under- and over-nutrition, micronutrient deficiency and marked socioeconomic disparity [5]. In addition, various nutritional and epidemiological transitions are occurring in LMICs; urbanisation, the transition from rural to urban lifestyles, is also likely to be contributing to changing patterns of disease and hip fracture incidence, through mechanisms such as reduced physical activity, increased trauma rates and changes in diet patterns. In addition, in LMIC settings, older people often play highly valued roles within their family as carers of younger generations, make economic contributions through continuing to work, and in turn, are often cared for by younger family members when necessary, impacting education and work of younger generations [11]. Therefore, the effects of fractures extend from an individual, to their family and potentially also the economy. Prevention strategies are therefore also likely to have wider effects. It is particularly alarming that the number of individuals at high risk of a major osteoporotic fracture has been predicted to double by 2040 worldwide [12]. This figure highlights the urgent need to better understand the epidemiology of musculoskeletal disease (including osteoporosis and sarcopenia) and fracture incidence, particularly in the ageing populations of LMICs with the aim of implementing prevention strategies.

## Fracture Incidence in Sub-Saharan Africa

Taking Sub-Saharan Africa (SSA) as an example of a LMIC region where fracture burden will rise dramatically over coming years, there are limited data. Notably, ethnic differences in hip fracture incidence have been reported in the first multi-centre, multi-ethnic study in South Africa across both the public and private healthcare sectors. Incidence rates, in adults aged 40 and above, were the highest in White and Indian men and women, and the lowest in Coloured and African groups. Importantly, Africans had the second-highest rate per capita. Women had higher rates than men except under 60-year olds [4••]. Further to this, in a sub-sample, 1 in 3 individuals died within a year of hip-fracture, comparable to the UK and the USA, and delays in surgery predicted mortality. African patients were more likely to die than Indian patients [13].

The prevalence of vertebral fractures was assessed in an opportunistic sample in a small study from South Africa, showing similar frequencies in Black (9%) and White (5%) women [14]. A similar vertebral fracture prevalence (9%) was observed in The Gambian Bone Ageing Study of 488 Gambians aged 40 years and over residing within 10 survey villages in rural Gambia; in addition, 3% of women and 0.4% of men self-reported hip fracture or fracture-like deformities [15].

Increasing evidence is therefore beginning to dispel the outdated myth that fragility fractures are not a problem in SSA. There remains limited evidence from across the regions, particularly in the most resource poor countries [5]. It is clear that data are needed to better understand underlying phenotypic differences that drive differences in fracture, and not to assume that risk factors in high-income countries apply to LMICs. This area should be the focus of research over coming years.

## Bone Densitometry

Various skeletal imaging methods are available, and include advanced techniques capable of estimating various parameters, in addition to bone mineral density. Dual-energy X-ray absorptiometry (DXA) is a 2-dimensional, projection technique which estimates areal BMD (aBMD); the fact that depth is not taken into account can lead to underestimation of aBMD of smaller bones, and overestimation in larger bones; therefore, in comparing populations or different groups of differing body size, this can lead to inaccuracies [16, 17]. Advantages of DXA are that it provides images of lateral spine for vertebral fracture assessment and detailed measures of body composition. In contrast, three-dimensional imaging techniques include peripheral quantitative computed tomography (pQCT) and high-resolution pQCT (HR-pQCT). Both methods are limited to peripheral skeletal sites, take physiological cross-sectional images, can provide separate measures of the cortical and trabecular compartments, and measure volumetric bone mineral density (vBMD), which is size-independent, bone size, shape and strength. In addition, HR-pQCT is also able to quantify bone microarchitecture parameters in the trabecular and cortical compartments such as trabecular number, thickness and plate properties and cortical porosity and cortical thickness. Finite element analysis (FEA) of HR-pQCT images is also possible using manufacturer software and provides extra detail of bone strength of each of the bone compartments. Further analysis of HR-pQCT data also includes individual trabecular segmentation (ITS), which characterises the plates and rods in the trabecular compartment [18].

It is well established that there are limitations to the assessment of fracture risk by DXA BMD alone, and that a low BMD does not necessarily predict fracture risk [19–21]. Enhancing phenotypic information to include more components of bone strength, such as bone shape, geometry and microarchitecture, may help to elucidate the reason for ethnic differences in fracture risk [21]. Therefore, although DXA is the clinical gold standard, and provides important insights into bone health, pQCT techniques may refine the understanding of differences in the cortical and trabecular compartments including microarchitecture and response to loading, to better define differences in ethnic bone phenotype and therefore fracture risk.

## Ethnic Differences in Bone Microarchitecture

A number of recent studies have investigated differences in bone microarchitecture between ethnic groups; an overview of these is given in Table 1. With the exception of one study from South Africa and another including India, all others are in high-income countries. There are no studies that currently directly determine the microarchitectural basis for ethnic differences in fracture prevalence or incidence. Though there are a growing number of studies providing evidence for aBMD and FRAX-risk independent associations between HR-pQCT outcomes and fracture, this remains to be explored in different ethnic groups [30–33].

**Table 1:** Key examples of heterogeneity between studies (published in the past 5 years) using pQCT or HR-pQCT to compare ethnic differences in the bone^a, b^

Analysis	Ethnic differences (direction of unadjusted differences, unless otherwise stated)	Differences at radius	Differences at tibia	
HR-pQCT in women	White American > Chinese-American and White American > Hong Kong Chinese (pre-menopausal)	Total area*		[22•]
	White American < Chinese-American and White American < Hong Kong Chinese (pre-menopausal)	Total bone density*, Ct. bone density*, Ct. thickness*, Tb. thickness*	Total bone density*, Ct. bone density*, Ct. thickness*, Tb. thickness*	
	White American > Chinese-American and White American > Hong Kong Chinese (post-menopausal)		Total area*, Tb. number*	
	White American < Chinese-American and White American < Hong Kong Chinese (post-menopausal)		Tb. separation*	
	Chinese > Caucasian (pre-menopausal)	Ct./total cross-sectional area, matrix mineral density*	Matrix mineral density*	[23]
	Caucasian > Chinese (pre-menopausal)	Total cross-sectional area, Ct. area, medullary cross-sectional area, Ct. porosity (total*, compact-appearing*, outer transitional zone)	Total cross-sectional area, Ct. area*, Medullary cross-sectional area*, Ct. porosity (total*, compact-appearing*, outer transitional zone*)	
	Chinese > Caucasian (post-menopausal)	Ct. porosity (inner transitional zone*)	Ct. porosity (inner transitional zone*)	
	Caucasian > Chinese (post-menopausal)	Total cross-sectional area, Ct. area*, medullary cross-sectional area*, Ct. thickness*, Whole bone stiffness*, Failure load*	Total cross-sectional area, Whole bone stiffness, Failure load	
pQCT in men	Black Afro-Caribbean > White European and South Asian, White European > South Asian	Ct. thickness*, Ct. area* (diaphysis)	Ct. area*, Ct. thickness, Cross-sectional area, CSMI (diaphysis)	[24]
	Rural South Indian < US Caucasian and Afro-Caribbean > US Caucasian	Ct. thickness (diaphysis)**	SSI (metaphysis)**	[25•]
	Rural South Indian<US Caucasian and Afro-Caribbean < US Caucasian		Ct. thickness (diaphysis)**	
	Rural South Indian>US Caucasian and Afro-Caribbean > US Caucasian		Endosteal circumference (diaphysis)**	
HR-pQCT in young adults	Caucasian > Asian men	Total bone area*, Tb. area*, Tb. number, Ct. porosity, Tb. stiffness, Whole bone stiffness, Whole bone failure load	Total bone area, Tb. area, Tb. number, Tb. stiffness, Whole bone stiffness, Whole bone failure load	[26]
	Asian > Caucasian men	Ct. density*, Ct. thickness*, Tb. heterogeneity*, Total density, Load-to-strength ratio	Ct. thickness	
	Caribbean Hispanic > non-Hispanic Caucasian men	Total density*, Ct. thickness*, Ct. area, Ct. density	Ct. density*, Ct. thickness*, Ct. area, Total density	[27]
	Non-Hispanic Caucasian > Caribbean Hispanic men	Total area*, Trabecular area*	Tb. area*, Ct. porosity*, Total area	
	White/Caucasian > Black/African-American men and women		Ct. porosity*, Tb. number* (metaphysis)	[28]
	Black/African-American > White/Caucasian men and women		Ct. area*, Total vBMD*, Ct. vBMD*, Ct. tissue mineral density*, Ct. thickness*, Tb. thickness*, Ct. area fraction*, Stiffness*, Failure load* (metaphysis)	[28]
	Black African > White African adolescent boys and girls	Trabecular vBMD	Total area, Ct.vBMD, SSI diaphysis	[29]
	White African > Black African adolescent boys and girls	Total area, Ct. crosssectional area, SSI (diaphysis)	Total area distal	[29]

*Ct* cortical, *Tb* trabecular, *CSM* cross-sectional moment of inertia, *SSI* stress strain index. All studies considered results at *p* < 0.05 statistically significant unless otherwise stated. *A significant difference also exists between the ethnic groups after various study-specific adjustments, **Adjusted results only presented, with results at *p* < 0.001 considered statistically significant

^a^The ethnic group definitions used in this table were those given by the author

^b^Where sexes are presented together either no sex-ethnicity interaction was found, or generalized results are presented for brevity

### Women

The most extensive HR-pQCT data are in comparisons of Chinese-American and Caucasian women. DXA-measured aBMD at the total hip, femoral neck and lumbar spine is lower in Chinese-American, compared to Caucasian, women across a range of age groups [34]. However, these differences in BMD do not necessarily translate to previous reports of fracture rates where Asians often have similar or lower risk of fracture [35] and studies with HR-pQCT have begun to explain this paradox [22, 23, 36–38]. In pre-menopausal women, both cortical and trabecular, thickness and vBMD were higher, and bone size smaller in Chinese-American women compared to White women [36]. Similar patterns were seen in post-menopausal women [37]. Further image analysis using ITS showed higher plate bone volume fraction and number which conferred greater bone strength in pre-menopausal Chinese-American women [18]. Further analyses of this cohort aimed to determine whether ethnic differences which exist in pre-menopausal women were also present in post-menopausal women, and whether post-menopausal differences reflected a different pattern of bone loss differ between ethnic groups. Data confirmed advantages in both trabecular and cortical bone in pre-menopausal Chinese women; in post-menopausal women, data suggest the cortical bone differences remain, with greater loss of trabecular bone with no ethnic differences in trabecular outcomes in the post-menopausal groups [38]. Furthermore, in a comparison of pre- and post-menopausal Chinese and Caucasian women in Australia, many of the ethnic differences seen in pre-menopausal women, such as lower total cortex porosity and higher plate bone volume fraction at the distal radius and tibia in Chinese women, were not observed when the same comparison was made in post-menopausal women [23]. In addition, differences in microarchitecture have been shown to exist in pre- and post-menopausal Chinese women in the USA and Hong Kong compared to White women in the USA, for example, plate to rod ratio at the tibia was higher in Chinese women (the USA and Hong Kong); however, this was not the case at the radius in post-menopausal women [22•]. This study helped to establish whether ethnic differences continued to exist across geographical locations.

Central QCT was used to determine differences in femoral neck geometry between post-menopausal Chinese women living in Beijing and Caucasian women living in Perth [39]. As previously reported, differences in bone mineral content were largely due to differences in body size; however, there was higher resistance to failure in Beijing, Chinese women due to wider femoral necks.

In African-American compared to Caucasian women, differences in bone microarchitecture, such as greater cortical thickness and area at the radius, have been shown [40]. In the same study, bone stiffness and failure load, from FEA, were also higher in African-American women [40]. Using ITS analysis, additional differences in trabecular microarchitecture have also been described; for example, plate bone volume fraction remained higher in African-American women, with this parameter also being a predictor of failure load at both sites [41•].

### Men

Recent studies have used pQCT to assess ethnic and geographic differences in bone phenotype. In men aged 40 and above from the UK, Black men had the highest DXA measured aBMD with far fewer differences in aBMD between White and South Asian men, despite White men having higher fracture risk than South Asian [6]. Similarly, using pQCT, Black men had larger bones, thicker cortices and greater vBMD. Cortical vBMD was the lowest in White men, though South Asians had smaller bones and lower distal vBMD than the other groups [24].

In a cross-cohort comparison between rural South Indian, the US Caucasian (MrOS) and Afro Caribbean (Tobago Cohort) men aged 60 and above, similar patterns were observed. Compared to the US-Caucasians, rural Indian men had lower bone strength, trabecular vBMD and cortical thickness, whereas Afro-Caribbeans’ had similar trabecular vBMD, higher cortical vBMD and greater strength [25•], similar to the reported UK differences [24]. More studies are required to understand how bone geometry translates to fracture risk in men.

### Adolescents and Young Adults

Finally, studies in young adults provide an important insight into when in the life course, ethnic differences in bone microarchitecture may be established. Such insights are likely to increase our understanding of the determinants of fracture risk later in life. Several studies published in recent years have used either pQCT, HR-pQCT or both, in younger adults around the time of, or before, the attainment of peak bone mass.

It has been shown that by young adulthood (in a study population with a mean age of 24.4 years), various differences in bone microarchitecture exist between Black African-American and White Caucasian men and women; interestingly, race and ethnic differences did not differ by sex [28]. Patterns were similar to those found elsewhere, with Black African American young men and women having greater cortical area, lower porosity, thicker trabeculae, greater trabecular vBMD and plate number density and surface area [28, 42]. This study indicates that ethnic differences in both cortical and trabecular bone microarchitecture are apparent by young adulthood.

Furthermore, in young adult men (with an age range of 25–35 years) of Asian compared to Caucasian ethnicity, size-adjusted DXA-measured aBMD at various sites did not differ between the ethnic groups. HR-pQCT revealed that differences in microarchitecture, including lower total bone area combined with greater cortical density and thickness in Asian men, remained at the radius. ITS analysis also revealed significant differences in microarchitecture at the radius including, as in women, higher plate-rod ratio, in the young Asian men [26]. It has also been shown that, although trabecular area was lower at the radius and tibia in Caribbean Hispanic, compared to non-Hispanic Caucasian, young men, smaller bone size was in combination with differences, notably in cortical bone microarchitecture, including greater cortical thickness at the radius and tibia and lower cortical porosity at tibia [27]. Both of these studies highlight adaptations, illustrated here in terms of microarchitecture, of smaller bones to maintain strength.

In South African adolescents, there were ethnic-specific differences in the radius and tibia at the end of growth where White adolescents had greater bone size and derived strength at the radius diaphysis, and Black adolescents greater size and strength at the tibia. At the end of growth, Black adolescents tended to have greater vBMD at the distal radius, the most common site of fracture [29].

## Conclusions

In conclusion, recent evidence concerning ethnic differences in fracture incidence and BMD, between and within countries, has been described. Furthermore, the need to increase our understanding of the picture of musculoskeletal disease and fracture risk in LMICs has been highlighted. Wherever ethnic differences in bone health exist, it is essential to fully understand their underlying causes. From this, there is the potential to design interventions and therefore reduce the healthcare burden associated with fracture.

In general, the results presented here indicate the ability of advanced imaging techniques to reveal cortical and trabecular differences in bone beyond the differences in bone mass which are highlighted by DXA. The use of pQCT imaging techniques, and their derived parameters, presents an opportunity to understand the potential contribution of bone microarchitecture in establishing bone strength. However, it is important to determine the underlying causes of these differences in microarchitecture, and wherever possible not to apply assumptions that one size fits all in understanding ethnic differences in fracture risk.

## Footnotes

1 In this review, we used ethnic group definitions given by the author.

2 Low- and middle-income countries are defined by the World Bank by their Gross National Income (GNI) per capita. Low income is GNI < $1026; low-middle $1026–3995; upper-middle $3996–12,375; high-income > $12,375
